# Influence of Grazing on Canola Grain, Canola Forage Yield, and Beef Cattle Performance

**DOI:** 10.3390/ani14030371

**Published:** 2024-01-24

**Authors:** Leonard M. Lauriault, Sangu V. Angadi, Glenn C. Duff, Eric J. Scholljegerdes, Murali K. Darapuneni, Gasper K. Martinez

**Affiliations:** 1 Rex E. Kirksey Agricultural Science Center, New Mexico State University, Tucumcari, NM 88401, USA; dmk07@nmsu.edu; 2 Agricultural Science Center, New Mexico State University, Clovis, NM 88101, USA; angadis@nmsu.edu; 3 Clayton Livestock Research Center, New Mexico State University, Clayton, NM 88415, USA; glennd@nmsu.edu; 4 Department of Animal and Range Sciences, New Mexico State University, Las Cruces, NM 88003, USA; ejs@nmsu.edu; 5 Agricultural Science Center, New Mexico State University, Farmington, NM 87401, USA; gasper@nmsu.edu

**Keywords:** canola, *Brassica napus*, grazing, grain, forage, beef cattle performance

## Abstract

**Simple Summary:**

Interest is increasing in grazing winter canola as an alternative crop in winter wheat rotations in the Southern High Plains of the USA and similar environments. In this study, winter cereal rye and winter canola pastures (forage) were compared for two winter growing seasons to determine the relative effect of pasture type on beef cattle performance, along with the effect of grazing on canola grain production. Canola grain yields were reduced by 25% when canola was grazed for approximately one month after grazing was initiated, but before the onset of rapid regrowth in spring. No differences existed for forage mass, nutritive value, or animal performance, although forage mineral composition of canola could be a concern. Grazing winter canola as a dual-purpose crop in semiarid environments is feasible when proper grazing management is applied; a 20–25% reduction in grain yield should be anticipated, but expect animal gains to offset that loss.

**Abstract:**

Interest is increasing in grazing winter canola (*Brassica napus*) as an alternative crop in winter wheat (*Triticum aestivum*) rotations in the Southern High Plains (SHP) of the USA and similar environments. In this stidy, winter cereal rye (*Secale cereale*) and winter canola pastures (forage) were compared for two winter growing seasons at New Mexico State University’s Rex E. Kirksey Agricultural Science Center at Tucumcari, NM, USA, to determine the relative effect of pasture type on late-gestation beef cows and growing yearling cattle, along with the effect of grazing on canola grain production. Canola grain yields were reduced by 25% when canola was grazed until removal approximately one month after grazing was initiated, but before the onset of rapid regrowth after winter (641 vs. 486 kg grain ha^−1^ for never grazed or grazed canola, respectively, *p* < 0.0256). No differences existed for forage mass, nutritive value, or animal performance, although forage mineral composition of canola could be a concern. Grazing winter canola as a dual-purpose crop in the SHP and similar environments is feasible when proper grazing management is applied; producers should anticipate a 20–25% reduction in grain yield, but expect animal gains to offset that loss.

## 1. Introduction

Interest is increasing in the use of winter canola (*Brassica napus*) as an alternative crop in winter wheat (*Triticum aestivum*) rotations in the Southern High Plains (SHP) of the USA, where wheat is grazed until it joints and cattle are removed to allow for grain production [1]. Including broadleaf crops in cereal grass rotations helps with soil health, increases weed and other pest control options, and mitigates wheat yield lag [2,3,4,5,6]. Additionally, the likelihood of winter survival is improved in the area compared to farther north [7]. A review of the existing literature indicates that many factors influence canola productivity, including seeding/stand density, environment, soil type, soil moisture availability and distribution, fertility, previous crop, tillage, and planting date [3].

Because winter forages for grazing are often in short supply in the SHP and similar environments [4], interest is also growing in whether canola can be grazed similarly to wheat as a dual-purpose crop with minimal effect on grain production [2,8]. Likewise, producers also are interested in the effect of grazing canola on beef cattle (*Bos taurus*) growth performance.

Dual-purpose forage/grain crops are already important components of integrated crop–livestock systems [1,9] and can produce equivalent grain yields to grain-only crops when appropriately managed in regard to animal health and nutrition, crop establishment and management, and the timing of grazing cessation to prevent crop reproductive damage [10]. Dual-purpose crops also can relieve grazing pressure on permanent pastures to allow for recovery and increased spring productivity [4,9] and, potentially, greater income for the farm system, even if the grazing period is only short-term during feed gaps between actively growing pastures [5]. Canola is already a value-added crop due to the extraction of the oil for cooking [1] or for biodiesel [6] and the subsequent use of the by-product meal as a valuable protein supplement for livestock industries [2,3].

As annual species, dual-purpose crops generally have greater nutritive value than perennial species, and that value is greatest when utilizing young growing stock intended for future sale rather than reproducing adults [5]; during the third trimester of pregnancy for spring calving beef cattle, nutritional requirements are greatest [11] because their unborn calf is rapidly growing at that time. Consequently, the availability of actively growing forage during that time with great nutritive value may offset more expensive supplementation when grazing lesser-quality forages, such as dormant native grasses [12]. Watt et al. [13] reported that dual-purpose canola and one other brassica had the greatest crude protein (CP) and metabolizable energy yields among 10 brassica genotypes and oat (*Avena sativa*) across seven site years in Australia, although the brassicas often had lesser forage mass production.

However, canola and other brassicas can accumulate nitrates [14] and cause bloating, potentially posing a health risk to grazing livestock [15]. Mineral imbalances also are common in actively growing crops other than wheat [15]. Additionally, many actively growing crops, such as canola, are high in water content and low in effective fiber but high in digestibility [2,14,15], thereby necessitating the provision of a lower-quality roughage to slow the ruminal passage of nutrients, although that has not been effective when grazing young wheat plants [15].

Similar to grazing winter wheat, appropriate grazing management is necessary to minimize any reduction in canola grain yield [4,10,16] as well as animal performance [14]. Under simulated grazing, canola and wheat forage dry matter (DM) yields were similar when harvested at a single time in late autumn, mid-winter, or early spring, about when wheat begins jointing and canola begins bud elongation [2]. While the forage nutritive value of canola was similar to or greater than wheat in regard to crude protein (CP) and neutral detergent fiber (NDF) in autumn and spring, it was slightly less in mid-winter due to leaf death and translocation of nutrients to storage components [2], as was available forage mass [17]. Under simulated grazing by clipping to near the ground level in late autumn, mid-winter, early spring, and late spring, the canola grain yield was reduced compared to a no-clipping treatment [2]. However, no actual grazing work has been performed in the southern Great Plains, where grazing canola can offer many benefits.

If grazing is feasible, canola could become an alternative dual-purpose value-added crop for the SHP and similar environments. To test its feasibility, the objectives of this study were to evaluate the influence of grazing on canola grain yield potential and to compare forage and animal performance when grazing canola compared to winter cereal forage, in this case, cereal rye (*Secale cereale*).

## 2. Materials and Methods

### 2.1. Site Description

Winter cereal rye and winter canola pastures (forage) were compared for two winter growing seasons (2016–17 and 2018–19) at New Mexico State University’s Rex E. Kirksey Agricultural Science Center at Tucumcari, NM, USA (35°12′0.5″ N, 103°41′12.0″ W; elev. 1247 masl) to determine the relative effect of pasture type on late-gestation beef cows and growing yearling cattle. Additionally, the effect of grazing on canola grain production was evaluated.

The climate in the region is Köppen–Geiger cold semiarid (http://www.cec.org/north-american-environmental-atlas/climate-zones-of-north-america/, accessed on 22 May 2023), characterized by cool, dry winters and warm, moist summers. Temperatures average 14.7° C and approximately 83% of the annual precipitation (398 mm annually) occurs as intermittent, relatively intense rainfall events from April to October. Weather data were collected from a station within 1 km of the study fields (Table 1).

### 2.2. Test Description and Management in 2016–17

The soils were Canez (fine-loamy, mixed, thermic Ustollic Haplargid) and Quay (fine-silty, mixed, superactive, thermic Ustic Haplocalcid) fine sandy loams. Pastures were 1.62 ha each in a completely randomized design with two replicates. The cereal rye pasture was a volunteer crop from the previous year. Canola was no-till planted into a flat seedbed on 26 August 2016 at 11.3 kg ha^−1^. Pastures were sprinkler-irrigated with treated municipal wastewater at rates shown in Table 1 to supplement precipitation. Irrigation was applied to canola only after cattle were removed each year to promote grain production. Nitrogen was applied on 17 January (14.6 kg N ha^−1^), 1 February (28 kg N ha^−1^), and 21 February 2017 (24.6 kg N ha^−1^).

Drought-induced shortage of young growing cattle for this study led to the use of privately owned late-gestation (>6 months), 3-year-old Angus cows (560 ± 60 kg), all having previously calved. Four animals were assigned to each pasture based on a combination of initial body weight, estimated months pregnant (6.9 ± 1.0), and body condition score (5.2 ± 0.5 on a 0 to 9 scale where 0 reflects emaciation and 9 reflects obesity). Bloat blocks (Sweetlix Bloat Guard^®^ Pressed Block; Ridley Block Inc., Mankato, MN, USA) and minerals (ADM Fall and Winter Beef Mineral, ADM, Quincy, IL, USA) were supplied ad libitum in each pasture. Additionally, based on initial forage nutritive value, the cattle were fed 1.1 kg/hd of Hi-Pro 20% Southwest Breeder Cubes (Hi-Pro Feeds, Friona, TX, USA) three times per week at the request of the owner. Hay was provided ad libitum in the canola pastures. Grazing was initiated on 15 December 2016 and ceased for canola pastures on 22 February 2017 and for cereal rye pastures on 2 March 2017, when the first calves were born for the respective treatments.

### 2.3. Test Description and Management in 2018–19

Winter canola and cereal rye pastures (1.62 ha each) were arranged in a randomized complete block design with three replicates in a different field in which the soil was Redona (fine-loamy, mixed, superactive, thermic Ustic Calciargid) fine sandy loam. Because the wastewater irrigation delivery system was off during the last half of August, land preparation was delayed and seed drilling into a flat seedbed took place from 26 to 28 September 2018 for canola and from 4 to 5 October 2018 for rye. Seeding rates were 39 and 11.3 kg ha^−1^ for rye and canola, respectively. As before, pastures were sprinkler-irrigated with treated municipal wastewater at rates shown in Table 1 to supplement precipitation and received 13.5 kg N ha^−1^ (5 December 2018) and 24.7 kg N ha^−1^ (22 January 2019) through the sprinkler. As in the 2016–17 study, irrigation was applied to canola only after cattle were removed each year to promote grain production.

Due to the lateness of planting, recently weaned beef cattle to be used on this trial were held at the New Mexico State University Clayton Livestock Research Center until 4 January 2019 when they were shipped to the study location and held on native grass pastures until the initiation of treatment grazing, which took place on 8 January. Prior to grazing, cattle were weighed (662 ± 48 lb body weight (BW)) and six steers were allocated to each pasture based on a near-uniform BW and standard deviation among pastures. Immediately prior to initiation of grazing and every 28 days thereafter until the steers were removed (13 March 2019), they were penned for 16 h and weighed individually on livestock scales. Minerals (ADM Fall and Winter Beef Mineral) were supplied ad libitum in each pasture and hay was continually available in the canola pastures.

### 2.4. Forage and Cattle Measurements

Prior to grazing, three 1.5 m^2^ exclosures were uniformly distributed in each pasture. Immediately prior to initiation of grazing and every 28 days thereafter, cattle were penned for 16 h, weighed, and standing forage was hand-clipped to ground level from a 0.4 m^2^ area near each exclosure. Sampling locations were selected to represent the standing forage in that area, but to avoid trampling adjacent to the exclosures. Harvested material from each sampling area was dried in a forced-air oven at 60 °C for 48 h to determine the dry matter (DM) mass. Initial forage samples were immediately delivered to Ward Laboratories (Kearney, NE, USA) for nutritive value estimation via near-infrared spectroscopy (NIRS) to determine any need for supplementation. Additional samples of canola were collected during grazing to monitor nitrate levels, which remained well within the safe category of <250 ppm (https://animalrangeextension.montana.edu/range/grazing-management/gm-nitrate-toxicity.html; accessed on 6 September 2023). The times between cattle weigh dates and weight differences were used to calculate average daily gains (ADG, kg hd^−1^ d^−1^) for the measurement periods.

### 2.5. Canola Grain Yield

To evaluate the impact of grazing cessation time on canola grain production, additional exclosures were installed near the original exclosures in the canola pastures on 17 January 2017 and 5 February 2019. After the cattle were removed from the canola pastures, the canola was left to grow with supplemental irrigation until sampling for final biomass and grain components was conducted within each exclosure on 22 June 2017 and 3 July 2019. For that sampling, whole plants were clipped (0.4-m^2^ area) to ground level and bagged carefully to minimize seed shattering. Plants were counted as they were clipped. After collection, the bag contents were poured into a large container and all pods that had not shattered in the bags were hand-threshed. Seed was passed through a screen to remove most inert material, after which the resultant seed was weighed.

### 2.6. Statistical Analysis

Forage mass and cattle data were averaged by pasture and analyzed using SAS Proc MIXED [18] to compare the effects of year, measurement period (Period), forage, and all possible interactions. The effect of time of grazing on final biomass and grain components was compared within the canola pastures. Forage replicates within the year were considered random. When the F-test for an interaction was significant (*p* < 0.05), lsmeans were separated using the PDMIX800 macro [19].

## 3. Results and Discussion

### 3.1. Canola Grain Variables

Canola grain yield was not influenced by year or the year × period interaction; however, despite grazing being excluded in mid-January (2016–17) or early February (2018–19), grain yields were reduced when canola was grazed until exclusion (641 vs. 486 kg grain ha^−1^ for never grazed or grazed canola, respectively, *p* < 0.0256). McGrath and his co-workers [10] also reported no treatment × year interactions for grain when canola or wheat was grazed. Irrigated canola grain yields in the present study were less than those reported nearby in the SHP (4360, 3040, 2940, 2720, and 930 kg ha^−1^ for never-clipped canola and for canola harvested for forage in late fall, mid-winter, early spring, and late spring, respectively) [2]. Canola grain yields in the high desert of northwestern New Mexico were 2393–5717 kg ha^−1^, depending on genotype [3] and the national average across environments and management regimes (1746 kg ha^−1^) [3].

Grain yield plant^−1^ was not influenced by grazing (55 g plant^−1^, *p* > 0.99). McCormick et al. [20] reported that grazing had no effect on pod number m^−2^, seed number m^−2^, or seed size compared to no grazing. During grazing in the present study, canola plant numbers decreased over measurement periods in 2018–19 (153 (initial), 137, and 91 (final) plants m^−2^ for periods 1 to 3, respectively, LSD_0.05_ = 53, *p* < 0.0695). A continued reduction in the number of plants was evident, in that, by grain harvest time, only 65 plants m^−2^ remained, but that was well within the target of 50–80 plants m^−2^ to maximize grain yield potential (https://www.canolacouncil.org/canola-encyclopedia/plant-establishment/seeding-rate/; accessed on 16 October 2023). Kirkegaard et al. [21] reported plant populations of >40 plants m^−2^ as being good and McCormick et al. [22] reported that 60–80 plants m^−2^ was a high plant density. Angadi et al. [23] reported that 40 plants m^−2^ did not reduce canola yield compared to 80 plants m^−2^ if the population was uniformly distributed, but yield was reduced if the 40 plants m^−2^ was not uniformly distributed.

Kirkegaard et al. [21] suggested that the greatest potential for canola as a dual-purpose crop would be in 450–650 mm precipitation areas, although its feasibility was also demonstrated in drier regions. The researchers [21] also reported that yields were reduced by a less favorable distribution of precipitation, particularly if dry weather occurred after grazing and limited the crop’s ability to recover [20]. While the present study environment received just under the long-term average of about 400 mm during the test years, irrigation was used to supplement precipitation, raising the total applied water (precipitation + irrigation) to >650 mm (Table 1).

Grazing-induced late maturity has been identified as a cause of reduced grain yields [21]; however, in the present study, there was no significant year effect when canola was grazed in either early or mid-winter. McCormick et al. [22] stated that post-flowering moisture and temperature stress limit grain yield [8]. Furthermore, McCormick et al. [20] associated reduced grain filling with hot conditions [6,8]. Additionally, weather data reported by McCormick et al. [20] indicated monthly average temperatures of approximately 12, 15, and 20 °C for the flowering through harvest period, making those hotter than average. Those temperatures are all below the actual readings in the present study and long-term averages for the same crop reproductive period (April to June) (Table 1). Consequently, the reproductive period temperature may have been a factor in low grain yield in the present study compared to elsewhere [6].

Nitrogen application timing and amount may also have been relevant factors [17], as Paye et al. [1] reported that 134 kg N ha^−1^ applied in spring or 50% of that applied in autumn and spring led to greater yields compared to autumn-only applications. The N applications in the present study (67.2 kg N ha^−1^ and 38.4 kg N ha^−1^ for 2016–17 and 2018–19, respectively) were considerably less, averaging 39% of the 134 kg N ha^−1^ used by Paye et al. [1]. Meanwhile, canola grain yields in the present study were 26% of those measured by Paye et al. [1], suggesting that multiple factors likely contributed to the differences between canola grain yields in the present study and those measured elsewhere. Sprague et al. [8] stated that canola requires approximately 80 kg N Mg^−1^ of grain. The average of 52 kg N ha^−1^ applied in the present study would, thus, support 654 kg ha^−1^ of grain production. Nutrient stress might also have affected canola recovery from grazing, but in the region of the present study, minimal external inputs are often used, especially in light of increased fertilizer prices.

The 11.3 kg ha^−1^ seeding rate also may have been a factor in grain production under grazing, as Stefanski et al. [14] reported that even at 5 kg ha^−1^, competition for light caused plants to elevate their growing point and receive damage from grazing. The researchers [14] stated that lower seeding rates, even as low as 2 kg ha^−1^, allowed space between plants that reduced hoof damage and kept the growing point below the grazing horizon. Cattle grazing preference was also greater for the lower seeding rate (2 kg ha^−1^) used by Stefanski et al. [14], likely because of a greater leaf-to-stem ratio due to the reduced competition. The prostrate growth habit of canola as a rosette is also expected to improve tolerance to winter-kill and some breeding programs have adopted that strategy develop dual-purpose winter canola (e.g., cv. Grffin [24]). Studies evaluating the grazing or clipping effects on canola grain yield and attaining greater yields used 4–6 kg ha^−1^ [2,4,10,17].

Begna et al. [2] reported no reduction in grain yield when canola was clipped to near the ground level in late autumn through to early spring in two years of a three-year study, but in one year, no grain was harvested when canola was clipped in mid-winter. Otherwise, clipping in late spring always reduced yield [2]. Kirkegaard et al. [21], in Australia, at a similar distance from the Equator as the present study, reported that the grain yield response to mowing to 10–15 cm in late July (January in the northern hemisphere) was about nil. However, grazing that either began or ceased on the same date reduced grain yields by 770 to 910 kg ha^−1^, grazing after the end of July led to >1000 kg ha^−1^ grain yield reduction, and grazing after flowering in September (March in the northern hemisphere) led to >60% decline in grain yield. Heavy grazing after July (December in the northern hemisphere) also reduced grain yields while light grazing did not [21].

While McGrath et al. [10] reported no reduction in wheat grain yields due to grazing, they did report a 20% reduction in canola grain yield, which is slightly less than the approximately 25% observed in the present study. Sprague et al. [8] observed similar results with decreases ranging from 25% to as much as 50%, depending on cultivar, when grazed in August (equal to February at the present study location). Consequently, producers should anticipate a 20–25% reduction in grain yields when canola is grazed either early (2018–19) or late (2016–17) during the dormant period.

### 3.2. Forage Mass and Nutritive Value

Forage mass also declined for both forages. There was no difference for the main effect of forage, but all interactions were significant for forage mass, including the highest-order interaction (Table 2). For the highest-order year × period × forage interaction, yields declined across 2016–17 when grazing took place earlier in the season (mid-December to mid-February), but not in 2018–19 when grazing was later (mid-January to mid-March) and included the initiation of active growth by both forages. In 2018–19, grazing ended before much spring growth could occur, which led to no difference across the period for either forage (Table 3). This, as well as soil type [21], likely contributed to the significant lower-order interactions and the trend (0.05 < *p* < 0.10 [25]) between years for forage mass (Table 2).

Previous research elsewhere [20] indicates that a total plant biomass of 5000 kg ha^−1^ at flowering is necessary for canola to maximize grain yield. Begna et al. [2] reported that winter canola has the potential to produce 5000 to 7000 kg ha^−1^ of forage for grazing during the autumn-to-early-spring period. McCormick et al. [22] found that grazing until mid-July (mid-January for the present study location) allowed sufficient time for biomass accumulation of 5000 kg ha^−1^ at flowering. The greatest forage mass in the present study occurred in December 2016 (Table 3) and was less than that reported by Begna et al. [2] in late spring (10,000 kg ha^−1^) when canola was at the bud elongation stage. Never attaining the 5000 kg ha^−1^ level likely influenced grain yield in the present study. Lauriault et al. [26], slightly north of the present study and at a much higher elevation, reported that brassicas had little autumn regrowth after being harvested 60 days after planting (DAP) in mid-August. The 60 d growth was slightly less than the 3120 kg ha^−1^ measure at 111 DAP in 2016–17 (Table 3). Nitrogen (50 kg ha^−1^) was applied at planting in the study by Lauriault et al. [26], while it was not applied until mid-January in 2016–17 of the present study. The cereal rye in 2016–17 of the present study had a similar initial forage mass to triticale (× *Triticosecale* Wittm. ex A. Camus (*Secale* × *Triticum*)) [12].

Kirkegaard et al. [21] reported that heavy grazing reduced grain yield compared to light grazing possibly because of the need for a longer recovery period after heavy grazing to regenerate leaf material for photosynthesis [17]. Grazing pressure in the present study was consistent with that applied to cereal forages in other studies at this location [12] and may have been heavy in regard to canola. Kirkegaard et al. [21] attributed lesser forage mass for grazed vs. mowed canola to a difference in defoliation, with more intensive defoliation under grazing due to selectivity of the more palatable leaf material. Prolonged grazing (>64 d) as used in the present study vs. short-duration grazing used by others (14 d) [21] may also have been a factor in recovery after grazing for grain production [21]. Kirkegaard et al. [21] stated that about 1.5 Mg forage mass ha^−1^ following grazing in mid–late July (January for the present study) was necessary for recovery and that grain yield penalties were likely if <1.0 Mg forage mass ha^−1^ was available after late July (January in the present study) because there would be insufficient time for recovery. Accordingly, very low levels of forage mass (<500 kg ha^−1^ [4]) after grazing both years in the present study (Table 3) may have contributed to the lower grain production, as previously described.

Winter dormancy begins with development of a plant rosette when temperatures drop below 1.7 °C, and leaf growth is stopped at −3.3 °C (https://webapp.agron.ksu.edu/agr_social/eu_article.throck?article_id=148, accessed on 11 October 2023). Winter temperature fluctuations, particularly in daily minimum temperatures, in the US Great Plains can trigger recurring dormancy and regrowth, leading to plant stress, although winter survival in the southern Great Plains, which includes the SHP, has the potential for 100% plant survival [7]. Djaman et al. [3] reported that canola was dormant from early November to early March in northwestern New Mexico while Paye et al. [1], about 75 km south of the present study, stated that canola was dormant between December and mid-February.

Although planting took place in late August and late September (February and March, respectively, in the southern hemisphere) in the present study in 2016 and 2018, respectively, canola was planted well before the time frame used by Kirkegaard et al. [21] in a planting date and grazing period study. Consequently, the canola in the present study would have been more well-established before the onset of dormancy with >90 d of growth between planting and dormancy [5]. Sprague et al. [8] reported reduced forage mass for grazing when canola was planted after early March (October in the present study). Kirkegaard et al. [21] reported that low plant numbers (≤2 plants m^−2^) were pulled out of the ground during early grazing periods (equivalent to December and January at the location of the present study in the northern hemisphere), but no plants were pulled out during later grazing periods. The earlier planting in the present study than in that by Kirkegaard et al. [21] likely allowed for greater root system development coupled with greater biomass before grazing in December 2016–17 (Table 3) and negated any plant uprooting. McCormick et al. [20] also reported that, as plant density increased from 27 to 67 plants m^−2^, early forage mass more than doubled. Hence, in the present study, plant population would not have limited forage production prior to grazing (Table 3).

Sprague et al. [17] found that canola had a greater growth rate than wheat prior to autumn grazing, which is consistent with the present study for canola and rye in 2016–17 (Table 3). The initial canola forage mass was also very similar to that reported by Sprague et al. [17], while in the rye yield, it was much less (average 3225 and 2350 kg ha^−1^ initial forage mass for canola and wheat, respectively [17]). When Sprague et al. [17] initiated grazing later (equivalent to late January in the present study), canola initial grazing mass was much less at 1800 kg ha^−1^. However, that was still much greater than the present study in 2018–19. In this regard, we must remember that theirs [17] was conducted in a much higher rainfall zone, with rainfall both before and during the grazing period, which was not matched even with irrigation applied in the present study (Table 1). This said, winter growth of canola is less than that of wheat [10].

While there was a trend (0.05 < *p* < 0.10 [25]) between years for initial forage CP, only the year × forage interaction was significant (Table 2 and Table 4) because rye was consistent across years, while canola had greater CP in 2018–19 than in 2016–17. The 2016–17 initial CP in the present study was similar to that measured at 60 DAP by Lauriault et al. [25] whether planted in mid-July or mid-August.

The difference within canola may have been due to the timing of when grazing began and the forage was sampled. In 2016–17, grazing began in December 2016, shortly after dormancy and leaf death but before leaves fell off. Begna et al. [2] reported that, after leaf death but before the leaves drop from the plant, nutrients are translocated to temporary storage locations and leaf nutritive value decreases. This does not happen with winter cereal forages, like wheat [2] or rye. The researchers [2] reported late autumn (rosette after dormancy, but before freeze damage), mid-winter (freeze-damaged leaves), early spring (included winter-damaged leaves and early spring growth), and late spring (50% flowering) CP of canola as 249–254, 181–194, 156–169, and 203–213 g kg^−1^, respectively. Canola CP in 2018–19 of the present study compares well with that measured by Begna et al. [2] and Stefanski et al. [14] for actively growing canola. Greater CP in late winter 2018–19 in the present study (Table 4) compared to that reported by Begna et al. [2] for early spring could be because winter-/freeze-damaged leaves were stripped off by weathering in the present study. Consequently, only new growth was available for grazing and sampling, as opposed to some leaves remaining attached at clipping in the Begna et al. [2] study [21], which would lead to a reduced/diluted CP concentration in the harvested forage.

In vitro true dry matter digestibility (IVTDMD) differed only between years (Table 2; 844 vs. 943 g kg^−1^ for 2016–17 and 2018–19, respectively). This is likely a result of the plant condition at the sampling time in relation to dormancy and freeze damage vs. the initiation of spring growth, as with CP. Masters and Thompson [15] reported digestibility >800 g kg^−1^ for both wheat and brassicas. Additionally, Begna et al. [2] reported no difference in NDF while Stefanski et al. [14] observed 660–730 g kg^−1^ rape (*Brassica napus*) digestibility over a range of seeding rates and grazing management.

Year, forage, and the year × forage interaction were all significant for Ca and the Ca:P ratio, but none were significant for P (Table 2). The interactions occurred due to a difference in the magnitude of increase in both forages from 2016–17 to 2018–19 (Table 4). Masters and Thompson [15] reviewed the literature and reported Ca concentrations of 7.1 and 4.2 g kg^−1^ for canola and triticale, respectively, which are double and similar to the Ca measured in the present study for canola and rye (a parent of triticale), respectively. For P, Masters and Thompson [15] reported 2.1 and 3.0 g kg^−1^ for canola and wheat, respectively. Their [15] canola P level was similar to canola in the present study, but their [15] wheat P level was 1.5× that of the cereal rye in the present study (Table 2). With greater Ca and lesser P, the Ca:P ratio is much greater than for other forages (0.91 for wheat and 3.38 for canola, as calculated from Masters and Thompson [15]) and was considerably greater in the present study (Table 2) than that calculated from Masters and Thompson [15].

McGrath et al. [10] stated that diversifying winter cropping systems to have multiple species (e.g., dual-purpose winter cereals and canola) available for grazing at any time [17] provides insurance against poor establishment and utilization of each species at the most optimum time for grain production. It also presents a crop rotation system that will help with integrated pest management and improve soil health [2,3,4,5]. Otherwise, grazing canola during the pre-dormancy vegetative stages is recommended to maximize nutritive value [10,14,17].

### 3.3. Animal Performance

No difference existed for either year or forage for ADG and there were no significant interactions; however, the period was significant with negative ADG during the first 28 d of grazing and positive ADG in the second 28 d (Table 2). The lack of any year effect when different classes of animals were used (third-trimester beef cows in 2016–17 vs. beef yearlings in 2018–19) likely occurred because the unborn calves were growing at about the same rate as the yearlings. Cattle fetuses grow on average from about 6 kg at the end of the second trimester to about 36 kg just before parturition [27]. Consequently, the ADG of the cow would average 0.33 kg d^−1^ during the third trimester [27]. Supplementation with the 20% breeder cubes would have supported fetal growth in 2016–17 for cows grazing canola since the canola had lesser CP that year, although the year × forage interaction was not significant for ADG (Table 2).

Regarding the negative ADG in the first grazing period each year (Table 2), Lauriault et al. [13] at the location of the present study, reported reduced animal performance in the first 28-d of grazing, likely due to acclimation to the environment and new forages. McCormick et al. [16] found that cattle spent 3 d grazing at fence lines before accepting the canola [14] and then attained ≥2 kg d^−1^ ADG. The researchers [16] also reported a lag phase in cattle weight gain that was likely about 2 weeks, but certainly less than the initial 28 d grazing period of the present study.

At any rate, it is quite possible that the ADG over 28 d for the second period (0.43 kg d^−1^) by 3.1 hd ha^−1^ (37 kg ha^−1^ total gain; calculated from Table 2) would have offset the loss of 155 kg ha^−1^ of canola grain due to grazing at the July grain price of USD 637.34 Mg^−1^ (https://albertacanola.com/daily-canola-prices/, accessed on 15 December 2023) (USD 98.79) and the USD 3.57 kg^−1^ March price for beef cattle (https://markets.businessinsider.com/commodities/live-cattle-price, accessed on 15 December 2023) for a total of USD 134.81. However, that does not address the negative gain during the initial 28 d of grazing, which was consistent across forage treatments in the present study (Table 2).

## 4. Conclusions

Based on the results of this study and the scientific literature, grazing winter canola as a dual-purpose crop in the SHP and similar environments is feasible when proper grazing management is applied. That involves early planting, the application of nitrogen at or shortly after planting and again after grazing is ceased, initiating grazing before or shortly after the onset of winter dormancy, and grazing lightly enough to protect the growing point. The performance when growing cattle-grazing winter canola was similar to that of winter cereals when availability was not limited. However, canola mineral composition should be monitored to maintain balances, and initial gains will likely be reduced for a brief period, after which compensation will likely occur. Producers should anticipate a 20–25% reduction in grain yield, but expect animal gains to offset that loss.

## Figures and Tables

**Table 1:** Monthly and annual mean air temperatures, total precipitation, and total irrigation at Tucumcari, NM, USA, during 2016–17 and 2018–19 and the long-term (1905–2022) means.

Year	July	Aug.	Sep.	Oct.	Nov.	Dec.	Jan.	Feb.	Mar.	Apr.	May	June	Annual
Temperature, C													
2016–17	27.8	25.0	22.8	18.9	11.1	3.9	3.3	8.9	13.3	14.4	17.8	25.0	16.0
2018–19	27.2	25.6	22.2	13.9	6.7	3.3	3.9	5.6	8.9	14.4	17.2	23.9	14.4
Long-term	26.2	25.2	21.6	15.2	8.6	4	3.5	5.6	9.5	14.2	19.1	24.3	14.7
Precipitation/irrigation, mm													
2016–17	40/25	59/54	10/44	0/54	35/0	9/19	26/22	4/38	55/89	69/38	46/51	25/51	380/295
2018–19	29/25	92/25	20/66	108/0	14/0	16/32	4/13	1/25	6/0	24/0	47/25	31/83	392/486
Long-term	67/---	68/---	39/---	34/---	17/---	16/---	10/---	12/---	19/---	28/---	47/---	47/---	398/---

**Table 2:** Means and results of statistical analysis for winter canola and cereal rye grazed in two years (2016–17 and 2018–19) at Tucumcari, NM, USA. Values are the lsmeans of two replicates in 2016–17 and three replicates in 2018–19.

	Period			Forage		*p*-Values						
	1	2	3	Canola	Rye	Year (Y)	Period (P)	Y × P	Forage (F)	Y × F	P × F	Y × P × F
FM, kg DM ha^−1^	1294 ^A^	584 ^B^	295 ^B^	820	629	0.0739	<0.0001	<0.0001	0.1808	0.0009	0.0201	0.0136
CP, g kg^−1^	-----	-----	-----	188	169	0.0736	-----	-----	0.3769	0.0205	-----	-----
IVTDMD, g kg^−1^	-----	-----	-----	919	909	0.0059	-----	-----	0.3276	0.9592	-----	-----
Ca, g kg^−1^	-----	-----	-----	14.20	4.38	0.0001	-----	-----	0.0001	0.0001	-----	-----
P, g kg^−1^	-----	-----	-----	2.01	1.95	0.3570	-----	-----	0.7717	0.5933	-----	-----
Ca:P	-----	-----	-----	7.17	2.37	0.0022	-----	-----	0.0002	0.0398	-----	-----
ADG, kg hd^−1^ d^−1^	-----	−0.22 ^B^	0.43 ^A^	0.18	0.03	0.1058	<0.0001	0.3647	0.1703	0.7559	0.7167	0.9462

FM, DM, CP, IVTDMD, Ca:P, and ADG signify forage mass, dry matter, crude protein, in vitro true DM digestibility, the calcium:phosphorus ratio, and average daily gains, respectively. Period means followed by different letters are significantly different at *p* < 0.05.

**Table 3:** The year × period × forage interaction (*p* < 0.0136) for forage mass of winter canola and cereal rye grazed in two years (2016–17 and 2018–19) at Tucumcari, NM, USA. Values are the lsmeans of two replicates in 2016–17 and three replicates in 2018–19.

Period	Canola	Rye
2016–17	kg ha^−1^	
Dec	3120 ^A^	1138 ^B^
Jan	963 ^BC^	610 ^BCD^
Feb	89 ^D^	176 ^D^
2018–19		
Jan	238 ^CD^	681 ^BCD^
Feb	289 ^CD^	472 ^BCD^
Mar	218 ^CD^	695 ^BCD^

Means within the interaction followed by similar letters are not significantly different at *p* < 0.05.

**Table 4:** The year × forage interaction for initial forage crude protein, calcium, and the calcium:phosphorus ratio (Ca:P) of canola and cereal rye grazed in two years (2016–17 and 2018–19) at Tucumcari, NM, USA. Values are the lsmeans of two replicates in 2016–17 and three replicates in 2018–19.

Period	Canola	Rye
Crude protein, g kg^−1^ (*p* < 0.0205)		
2016–17	138 ^B^	179 ^AB^
2018–19	239 ^A^	161 ^B^
Calcium, g kg^−1^ (*p* < 0.0001)		
2016–17	10.1 ^B^	3.5 ^D^
2018–19	18.3 ^A^	5.3 ^C^
Ca:P (*p* < 0.0398)		
2016–17	4.93 ^B^	1.65 ^C^
2018–19	9.41 ^A^	3.10 ^BC^

Means within an interaction followed by similar letters are not significantly different at *p* < 0.05.

## Data Availability

Data are available upon reasonable request from the authors.
